# Limited Early Warnings and Public Attention to Coronavirus Disease 2019 in China, January–February, 2020: A Longitudinal Cohort of Randomly Sampled Weibo Users

**DOI:** 10.1017/dmp.2020.68

**Published:** 2020-04-03

**Authors:** Yuner Zhu, King-Wa Fu, Karen A. Grépin, Hai Liang, Isaac Chun-Hai Fung

**Affiliations:** Journalism and Media Studies Centre, The University of Hong Kong, Hong Kong; School of Public Health, The University of Hong Kong, Hong Kong; School of Journalism and Communication, The Chinese University of Hong Kong, Hong Kong; Jiann-Ping Hsu College of Public Health, Georgia Southern University, Statesboro, GA

**Keywords:** awareness, coronavirus, health communication, social media, Weibo

## Abstract

**Objective:**

Awareness and attentiveness have implications for the acceptance and adoption of disease prevention and control measures. Social media posts provide a record of the public’s attention to an outbreak. To measure the attention of Chinese netizens to coronavirus disease 2019 (COVID-19), a pre-established nationally representative cohort of Weibo users was searched for COVID-19-related key words in their posts.

**Methods:**

COVID-19-related posts (*N* = 1101) were retrieved from a longitudinal cohort of 52 268 randomly sampled Weibo accounts (December 31, 2019–February 12, 2020).

**Results:**

Attention to COVID-19 was limited prior to China openly acknowledging human-to-human transmission on January 20. Following this date, attention quickly increased and has remained high over time. Particularly high levels of social media traffic appeared around when Wuhan was first placed in quarantine (January 23–24, 8–9% of the overall posts), when a scandal associated with the Red Cross Society of China occurred (February 1, 8%), and, following the death of Dr Li Wenliang (February 6–7, 11%), one of the whistleblowers who was reprimanded by the Chinese police in early January for discussing this outbreak online.

**Conclusion:**

Limited early warnings represent missed opportunities to engage citizens earlier in the outbreak. Governments should more proactively communicate early warnings to the public in a transparent manner.

Since December 2019, the world witnessed an epidemic of the severe acute respiratory syndrome coronavirus 2 (SARS-CoV-2). After cases of coronavirus disease 2019 (COVID-19) appeared in the city of Wuhan, local health authorities made a public announcement and China notified the World Health Organization about the outbreak on December 31, 2019.^1^ In a matter of weeks, tens of thousands of cases would appear throughout China and in many countries around the world.

In a tightly controlled media environment, it is unclear when regular Chinese citizens became aware of the outbreak and if aware, how much attention they paid to it. Awareness and attentiveness may have implications for the acceptance and adoption of prevention and control measures. To measure the attention of Chinese netizens to COVID-19, we used a pre-established nationally representative cohort of randomly sampled Weibo users and searched for COVID-19-related key words in their posts.

## METHODS

Sina Weibo is the largest microblog service provider in mainland China, that is, China’s equivalent of Twitter. The social media platform had 497 million monthly active users as of September 2019.^2^ The data used herein were collected by Weiboscope, a research project led by coauthor KWF. Since 2010, the research team has been collecting the social media data through Sina Weibo’s Open Application Programming Interface by sampling a list of high-profile users and random users, whose posts are programmatically retrieved every 15–20 minutes by a cluster of computer servers.^3^ If a once-published post is found to be absent in the next retrieval of the user’s timeline, an additional request is made to confirm whether the post is truly censored, that is, return of an error message of “permission denied” indicating a censored message.^3^

The current study analyzed the posts from 52 268 randomly sampled accounts in the Weiboscope database, whose 10-digit unique user identity codes were randomly selected in 2015 and their published posts have been longitudinally recorded. This cohort constitutes a representative sample of the whole user population of Weibo.^4^

We compiled *a priori* a list of key words in simplified Chinese characters that were pertinent to the COVID-19 outbreak in China. The key word list was checked and confirmed by all authors, including bilingual epidemiologists and communication scientists, to be sufficiently inclusive and specific (see Supplementary Technical Appendix).

**FIGURE 1:**
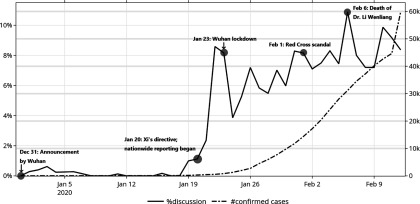
Time Series of Percentage of Daily Weibo Posts Pertinent to COVID-19 Posted by a Cohort of Randomly Sampled Weibo Users, and the Daily Cumulative Number of Confirmed Cases From December 31, 2019, Through February 12, 2020.

The time frame of our study was from December 31, 2019 through February 12, 2020. A daily percentage was computed by dividing the daily total number of COVID-19-related posts by the daily total number of published posts in the samples (Table 1, Figure 1). Daily cumulative counts of confirmed COVID-19 cases in mainland China were obtained from press releases published by the National Health Commission, Wuhan Municipal Health Commission, and Guangdong Provincial Health Commission. Both daily series were plotted on a time trend diagram (see Figure 1).

**TABLE 1:** Number and Percentage of COVID-19-Related Weibo Posts With Selected Key Words

Key Word	English Translation	Number^*^	Percentage^**^
疫情	epidemic situation	383	34.79%
口罩	mask	259	23.52%
病毒	virus	230	20.89%
肺炎	pneumonia	191	17.35%
冠状	coronavirus	132	11.99%
感染	infected	116	10.54%
确诊	confirmed case	100	9.08%
隔离	quarantine	66	5.99%
防疫	combat the outbreak	38	3.45%
传染	infection	36	3.27%
新冠	new coronavirus	36	3.27%
钟南山	Zhong Nanshan	30	2.72%
封城	lockdown	28	2.54%
非典	SARS	27	2.45%
N95	N95	26	2.36%
李文亮	Li Wenliang	26	2.36%
蝙蝠	bat	19	1.73%
防护服	hazmat suit	17	1.54%
卫健委	health commission	11	1.00%
世卫	WHO (abbreviated)	10	0.91%
重症	severe	8	0.73%
疾控中心	Center(s) for Disease Control and Prevention	7	0.64%
李兰娟	Li Lanjuan	7	0.64%
流行病	epidemiology	6	0.55%
华南海鲜市场	Huanan Seafood Market	5	0.45%
人传人	human-to-human transmission	5	0.45%
管轶	Guan Yi	4	0.36%
世界卫生组织	World Health Organization	4	0.36%
消毒液	bleach	3	0.27%
洗手液	hand sanitizer	3	0.27%
危重	critically ill	3	0.27%
张文宏	Zhang Wenhong	3	0.27%
CDC	Center(s) for Disease Control and Prevention	3	0.27%
高福	Gao Fu (George F. Gao)	2	0.18%
穿山甲	pangolin	2	0.18%
粪口传播	fecal–oral transmission	2	0.18%
WHO	World Health Organization	2	0.18%
飞沫传播	droplets transmission	2	0.18%
疑似病例	suspected case	2	0.18%
潜伏期	incubation period	1	0.09%

* Key words may be occurring in the same piece of posts. Therefore, the totals in the numbers column will exceed 1101 and the totals in percentages column will exceed 100%.

** Percentage of the population of posts identified with at least 1 of the given key words (N = 1101).

## RESULTS AND DISCUSSION

A total of 1101 Weibo posts pertinent to COVID-19 were identified. Table 1 shows the key word frequency in the published posts made by the random user samples in the study period. We found little evidence that Chinese netizens paid much attention to the outbreak before January 20 when the Chinese Government for the first time openly acknowledged that human-to-human transmission of the virus was happening and when China initiated nationwide reporting on the outbreak.^5,6^ Our data show that, following this date, attention to the outbreak quickly increased and has remained high over time. Particularly high levels of social media traffic also appeared around when Wuhan was first placed in quarantine (January 23–24, 8–9% of the overall posts), when a scandal associated with the Red Cross Society of China occurred (February 1, 8%), and, following the death of Dr Li Wenliang (February 6–7, 11%), 1 of the whistleblowers who was reprimanded by the Chinese police in early January for discussing this outbreak on WeChat groups.^7^ See Technical Appendix for sample posts for each of these events.

### Limitations

This study has its limitations. First, our data collection system is not able to capture social media posts that were filtered by Sina Weibo’s interface or disappeared within the data retrieval intervals. Only 8 post-publication censored posts were detected among the random samples in the study period. The authors cannot completely rule out the impact of Internet censorship in China. Second, Weibo is only 1 of several popular social media platforms in mainland China. This study does not cover other platforms, such as WeChat.

## CONCLUSION

Low levels of attention to the outbreak among Chinese citizens in early January may represent a missed window of opportunity to contain the outbreak. Given that the adoption of personal protective behaviors has been shown to be associated with trust in government and that large-scale social distancing measures have been put in place in many parts of China,^8,9^ ensuring that citizens are aware of the true severity of the outbreak in its early stages is likely to increase acceptance and compliance with prevention and control measures. Governments worldwide should take note of lessons learned in China and should more proactively communicate early warnings to the public in a transparent manner.

## Supplementary material

For supplementary material accompanying this paper visit http://dx.doi.org/10.1017/dmp.2020.68. click here to view supplementary material
