# Supplementary material for: Limited Early Warnings and Public Attention to Coronavirus Disease 2019 in China, January–February, 2020: A Longitudinal Cohort of Randomly Sampled Weibo Users
Source: Disaster Med Public Health Prep. 2020 Apr 3:1–4. doi: 10.1017/dmp.2020.68 (PMC7171227; doi:10.1017/dmp.2020.68)
Supplement: Supplementary file 1 [file S1935789320000683sup001.docx]

**Limited early warnings and public attention to COVID-19 in China, January-February, 2020: a longitudinal cohort of randomly sampled Weibo users**

**Technical Appendix**

Authors: Yuner Zhu^1^, King-Wa Fu^1^, Karen A. Grépin*^2^, Hai Liang^3^, Isaac Chun-Hai Fung*^4^

Affiliations:

^1^ Journalism and Media Studies Centre, The University of Hong Kong

^2^ School of Public Health, The University of Hong Kong

^3^ School of Journalism and Communication, The Chinese University of Hong Kong

^4^ Jiann-Ping Hsu College of Public Health, Georgia Southern University

* Both Dr. Karen A. Grépin and Isaac Chun-Hai Fung serve as corresponding authors. Correspondence should be addressed to: Karen A. Grépin, School of Public Health, Li Ka Shing Faculty of Medicine, The University of Hong Kong, Pokfulam, Hong Kong. Telephone: +852 39179719. Email: [kgrepin@hku.hk](mailto:kgrepin@hku.hk); Isaac Chun-Hai Fung, Department of Biostatistics, Epidemiology & Environmental Health Sciences, Jiann-Ping Hsu College of Public Health, Georgia Southern University, Statesboro, GA 30460-7989. Telephone: +1 912 478 5079. Email: [cfung@georgiasouthern.edu](mailto:cfung@georgiasouthern.edu)

**Technical Appendix**

**List of keywords and their frequencies in the dataset**

Weibo posts pertinent to COVID-19 were identified according to a set of predefined keywords in simplified Chinese characters:

1. ‘疫情’ epidemic situation,
2. ‘口罩’ mask,
3. ‘病毒’ virus,
4. ‘肺炎’ pneumonia,
5. ‘冠状’ corona(virus),
6. ‘感染’ infected,
7. ‘确诊’ confirmed case,
8. ‘隔离’ quarantine,
9. ‘防疫’ combat the outbreak,
10. ‘传染’ infection,
11. ‘新冠’ new coronavirus,
12. ‘钟南山’ Zhong Nanshan,
13. ‘封城’ lockdown,
14. ‘非典’ SARS,
15. ‘N95’ N95,
16. ‘李文亮’ Li Wenliang,
17. ‘蝙蝠’ bat,
18. ‘防护服’ hazmat suit,
19. ‘卫健委’ health commission,
20. ‘世卫’ WHO (abbreviation for the World Health Organization),
21. ‘重症’ severe,
22. ‘疾控中心’ CDC (abbreviation for centers for disease control and prevention, of China or the US or elsewhere),
23. ‘李兰娟’ Li Lanjuan,
24. ‘流行病’ epidemiology,
25. ‘华南海鲜市场’ Huanan Seafood Market,
26. ‘人传人’ human-to-human transmission,
27. ‘管轶’ Guan Yi,
28. ‘世界卫生组织’ WHO,
29. ‘消毒液’ bleach,
30. ‘洗手液’ hand sanitizer,
31. ‘危重’ critically ill,
32. ‘张文宏’ Zhang Wenhong,
33. ‘CDC’ CDC,
34. ‘高福’ Gao Fu (i.e., George F. Gao, Director of China CDC),
35. ‘穿山甲’ pangolin,
36. ‘粪口传播’ fecal–oral transmission,
37. ‘WHO’ WHO,
38. ‘飞沫传播’ droplets transmission,
39. ‘疑似病例’ suspected case,
40. ‘潜伏期’ incubation period.

**Sample Weibo Posts**

Here below we select three sample Weibo posts to illustrate the three events mentioned in the main text.

1. **On January 23, 2020: Wuhan “lockdown”**

“早起看到武汉封城的消息，看来情况是很严重了，再看看丁香园的数据，一夜过去，又多了几百例新发和几例死亡，数据攀升之快，令人震惊！\n仔细看了死亡病例的简要病史，总结了下，有以下几个特点：高龄、合并基础疾病（尤其是糖尿病和慢阻肺）、出现呼吸道症状后迅速进展为呼吸困难氧饱和度下降、胸部CT...”

(English Translation: I got up early this morning to see the news of Wuhan lockdown. It seems the situation is really bad there. Then I saw the data reported by DingXiangYuan. A few hundred new cases and several new deaths were added overnight. It’s shocking that the stats are rising so fast! I closely read clinic records of death cases and summarize their shared characteristics as follows: aged, with underlying health conditions (especially diabetes and chronic obstructive pulmonary disease), quickly developing into breathing difficulty and low blood oxygen after the onset of pneumonia symptoms), chest CT…)

1. **On February 1, 2020: A scandal associated with the Red Cross Society of China**

“事情不分轻重缓急吗？你们如果给其他定点医院1.6万个口罩，给仁爱医院3000个，我们会骂你们吗？？办事不会过脑子吗？？仁爱医院都能拿出口罩免费发给社区居民，说明医院就不是非常紧缺物资，你们还发那么多，怎么想的？？怎么办事的？？？怎么不该骂？！！”

(English Translation: Do you know how to prioritize your work? Will we scold you if you give 16 thousand masks to designated hospitals and give 3 thousand masks to Ren’ai Hospital? Can you use your brain to think while you are doing your work? Ren’ai Hospital is even capable of giving out masks to the neighborhood free of charge, which means they are not short of medical supplies. Why do you still give them so many masks? What is in your brain? How could you do so??? Are you supposed to be scolded?)

1. **On February 6, 2020: The death of Dr. Li Wenliang**

“#李文亮医生去世#我觉得普通工作者在大是大非的问题上能坚守自己的三观尤为可贵。仅仅因为在其职，就不得已去行有违三观之事，实在是煎熬的。到头来即使内心最终平复了，也怕是识不得自己的样子了。比如训诫的警察同志，比如操作降热搜的新浪工作人员。”

(English Translation: #Dr. Li Wenliang Died# I think it is most precious that normal people can insist their principles on the fundamental issues. It will be really suffering for one if he or she has to run counter to their own principles simply because of their jobs. If so, at the end of the day, even if we could find our inner peace, we will turn ourselves into another person that we cannot recognize. For example, the policemen who admonish people, and the moderators who work for Sina and try to degrade the popularity of public discussions.)

References

[1 . Weibo Corporation. Weibo Reports Third Quarter 2019 Unaudited Financial Results. 2019; published online Nov 14. https://weibocorporation.gcs-web.com/news-releases/news-release-details/weibo-reports-third-quarter-2019-unaudited-financial-results (accessed Feb 14, 2020).](https://www.zotero.org/google-docs/?FiGrYp)

[2 . Fu KW, Chan C, Chau M. Assessing censorship on microblogs in China: Discriminatory keyword analysis and the real-name registration policy. *IEEE Internet Comput* 2013; **17**: 42–50.](https://www.zotero.org/google-docs/?FiGrYp)

[3. Fu KW, Chau M. Reality check for the Chinese microblog space: a random sampling approach. *PloS One* 2013; **8**: e58356–e58356.](https://www.zotero.org/google-docs/?FiGrYp)
